# Complete genome sequence of *‘Thermobaculum terrenum’* type strain (YNP1^T^)

**DOI:** 10.4056/sigs.1153107

**Published:** 2010-10-27

**Authors:** Hajnalka Kiss, David Cleland, Alla Lapidus, Susan Lucas, Tijana Glavina Del Rio, Matt Nolan, Hope Tice, Cliff Han, Lynne Goodwin, Sam Pitluck, Konstantinos Liolios, Natalia Ivanova, Konstantinos Mavromatis, Galina Ovchinnikova, Amrita Pati, Amy Chen, Krishna Palaniappan, Miriam Land, Loren Hauser, Yun-Juan Chang, Cynthia D. Jeffries, Megan Lu, Thomas Brettin, John C. Detter, Markus Göker, Brian J. Tindall, Brian Beck, Timothy R. McDermott, Tanja Woyke, James Bristow, Jonathan A. Eisen, Victor Markowitz, Philip Hugenholtz, Nikos C. Kyrpides, Hans-Peter Klenk, Jan-Fang Cheng

**Affiliations:** 1 Los Alamos National Laboratory, Bioscience Division, Los Alamos, New Mexico, USA; 2 ATCC- American Type Culture Collection, Manassas, Virginia, USA; 3 DOE Joint Genome Institute, Walnut Creek, California, USA; 4 Biological Data Management and Technology Center, Lawrence Berkeley National Laboratory, Berkeley, California, USA; 5 Oak Ridge National Laboratory, Oak Ridge, Tennessee, USA; 6 DSMZ - German Collection of Microorganisms and Cell Cultures GmbH, Braunschweig, Germany; 7 Thermal Biology Institute, Montana State University, Bozeman, Montana, USA; 8 University of California Davis Genome Center, Davis, California, USA

**Keywords:** extreme thermal soil, thermoacidophile, Gram-positive, nonmotile, non-spore-forming, obligate aerobe, *Incertae sedis*, *Chloroflexi*, GEBA

## Abstract

*‘Thermobaculum terrenum’* Botero *et al.* 2004 is the sole species within the proposed genus *‘Thermobaculum’*. Strain YNP1^T^ is the only cultivated member of an acid tolerant, extremely thermophilic species belonging to a phylogenetically isolated environmental clone group within the phylum *Chloroflexi*.  At present, the name *‘Thermobaculum terrenum’* is not yet validly published as it contravenes Rule 30 (3a) of the Bacteriological Code. The bacterium was isolated from a slightly acidic extreme thermal soil in Yellowstone National Park, Wyoming (USA). Depending on its final taxonomic allocation, this is likely to be the third completed genome sequence of a member of the class *Thermomicrobia* and the seventh type strain genome from the phylum *Chloroflexi.* The 3,101,581 bp long genome with its 2,872 protein-coding and 58 RNA genes is a part of the *** G****enomic* *** E****ncyclopedia of* *** B****acteria and* *** A****rchaea * project.

## Introduction

Strain YNP1^T^ (= ATCC BAA-798 = CCMEE 7001) is the proposed type strain of the not yet validly published species ‘*Thermobaculum terrenum’*, which represents the type species of the not yet validly published genus name *‘Thermobaculum’* [1]. The strain was cultivated from a moderately acidic (pH 3.9) extreme thermal soil in Yellowstone National Park (YNP), Wyoming (USA) for which a thorough chemotaxonomic characterization was published by Botero *et al.* in 2004 [1]. Although the biological characteristics of the novel strain fulfill all criteria required for the type strain of a novel genus, the proposed name *‘Thermobaculum terrenum’* (= hot small rod belonging to earth/soil) has not yet been validly published (= included in one of the updates of the Validation List that is regularly published in *Int J Syst Evol Bacteriol*), because rule 30 (3a) of the Bacteriological Code (1990 Revision), which requires that as of 1^st^ January 2001 the description of a new species [...] must include the designation of a type strain, and a viable culture of that strain must be deposited in at least two publicly accessible service collections in *different* countries from which subcultures must be available [2]. Strain YNP1^T^ is currently deposited only in two US culture collections. Here we present a summary classification and a set of features for *‘T. terrenum’* strain YNP1^T^, together with the description of the complete genomic sequencing and annotation.

## Classification and features

Based on analyses of 16S rRNA gene sequences, strain YNP^T^ is the sole cultured representative of the genus ‘*Thermobaculum*’. It has no close relatives among the validly described species within the *Chloroflexi*. The type strain of *Sphaerobacter thermophilus* [3] shares the highest pairwise similarity (84.9%), followed by *Thermoleophilum album* and *T. minutum* [4-6], the two sole members of the actinobacterial order *Thermoleophilales* [7] with 83.6% sequence identity, and three type strains from the clostridial genus *Thermaerobacter* (83.2-83.5%) [8], that are currently not placed within a named family. Only four uncultured bacterial clones in GenBank share a higher degree of sequence similarity with strain YNP^T^ than the type strain of the ‘closest’ related species, *S. thermophilus*. These are clone DRV-SSB031 from rock varnish in the Whipple Mountains, California (92.1%) [9], and clones AY6_14 (FJ891044), AY6_27 (FJ891057) and AY6_18 (FJ891048) from quartz substrates in the hyperarid core of the Atacama Desert (86.9-87.9%). No phylotypes from environmental screening or metagenomic surveys could be linked to ‘*T. terrenum’*, indicating a rather rare occurrence in the habitats screened thus far (as of September 2010). A representative genomic 16S rRNA sequence of ‘*T. terrenum’* YNP^T^ was compared using BLAST with the most recent release of the Greengenes database [10] and the relative frequencies of taxa and keywords, weighted by BLAST scores, were determined. The three most frequent genera were *Thermobaculum* (81.2%), *Sphaerobacter* (10.3%) and *Conexibacter* (8.4%). The five most frequent keywords within the labels of environmental samples which yielded hits were 'microbial' (3.6%), 'waste' (3.3%), 'soil' (3.3%), 'simulated' (3.2%) and 'level' (3.1%). The five most frequent keywords within the labels of environmental samples which yielded hits of a higher score than the highest scoring species were 'soil' (4.5%), 'structure' (3.3%), 'simulated' (3.2%), 'level/site/waste' (2.9%) and 'core' (2.1%).

**Figure 1:**
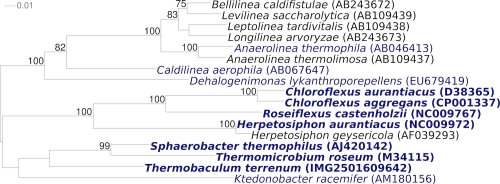
Phylogenetic tree highlighting the position of *‘T. terrenum’* strain YNP^T^ relative to the type strains of the other species within the phylum *Chloroflexi* . The trees were inferred from 1,316 aligned characters [11,12] of the 16S rRNA gene sequence under the maximum likelihood criterion [13] and rooted in accordance with the current taxonomy. The branches are scaled in terms of the expected number of substitutions per site. Numbers above the branches are support values from 1,000 bootstrap replicates [14] if larger than 60%. Lineages with type strain genome sequencing projects registered in GOLD [15] are shown in blue, published genomes [16] and GenBank records [CP000804,CP000875,CP000909,CP001337] in bold, *e.g.* the GEBA genome *S. thermophilus* [17].

Figure 1 shows the phylogenetic neighborhood of ‘*T. terrenum’* strain YNP^T^ in a 16S rRNA based tree. The sequences of the two identical 16S rRNA gene copies in the genome do not differ from the previously published 1,333 nt long partial sequence generated from ATCC BAA-798 (AF391972).

**Figure 2:**
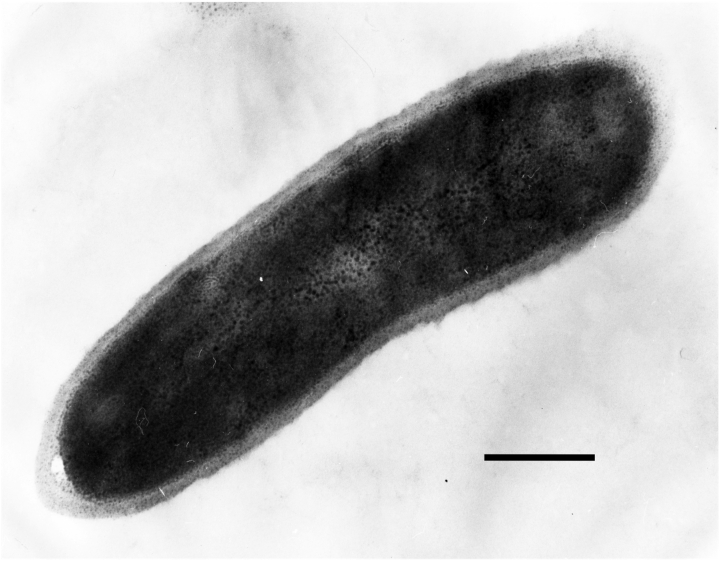
Transmission electron micrograph of ‘*T. terrenum’* strain YNP1^T^, scale bar 0.1 μm

The cells of strain YNP1^T^ are 1-1.5 × 2-3 μm long, non-motile rods (Figure 2 and Table 1), enveloped by a thick cell wall external to a cytoplasmic membrane [1]. YNP1^T^ cells occur singly or in pairs, stain Gram-positive in the exponential growth-phase, are obligately aerobic, and non-spore-forming [1]. Colonies are pink-colored and growth occurs best at pH 6-8 (pH_opt_ 7) and 67°C, with a possible temperature range of 41-75°C [1]. Culture doubling time at *T*_opt_ was 4 hours and increases sharply above 70°C, whereas growth at the temperature extremes was relatively poor [1]. Cells grow best in complex media containing 0.5% NaCl and yeast extract (for growth factors) [1], but also on sucrose, fructose, glucose, ribose, xylose, sorbitol, and xylitol [1]. Strain YNP1^T^ was positive for catalase, urease, and nitrate reduction, but tested negative for oxidases, and was also negative for fermentation of glucose or lactose [1]. No anaerobic growth was observed in the presence of sulfate, nitrate, ferric iron, or arsenate as possible electron acceptors [1]. No chemolithoautotrophic growth was observed in an experimental matrix that included the electron donors H_2_, H_2_S, or S_0_ with oxygen as the electron acceptor. Surprisingly, the *in vitro* pH optimum of strain YNP1^T^ (pH 7) is much higher than that of the soil from which it was isolated (pH 4-5) [1]. In pure culture, strain YNP1^T^ failed to grow at such low pH values, suggesting that the thermal soil habitat is not optimal for the strain [1].

**Table 1:** Classification and general features of ‘*T. terrenum’* strain YNP1^T^ according to the MIGS recommendations [18].

MIGS ID	Property	Term	Evidence code
	Current classification	Domain *Bacteria*	TAS [19]
		*Phylum Chloroflexi*	TAS [20-23]
		Class *Incertae sedis*	NAS
		Order *Incertae sedis*	NAS
		Family *Incertae sedis*	NAS
		Genus *‘Thermobaculum’*	TAS [1]
		Species *‘Thermobaculum terrenum’*	TAS [1]
	Type strain	YNP1	TAS [1]
	Gram stain	positive	TAS [1]
	Cell shape	short rods (1-1.5 × 2-3 µm)	TAS [1]
	Motility	non-motile	TAS [1]
	Sporulation	none	TAS [1]
	Temperature range	65°C-92°C	TAS [1]
	Optimum temperature	67°C	TAS [1]
	Salinity	0.5% NaCl	TAS [1]
MIGS-22	Oxygen requirement	obligate aerobic	TAS [1]
	Carbon source	glucose, fructose, ribose, sorbitol, sucrose, xylose, xylitol	TAS [1]
	Energy source	heterotrophic	TAS [1]
MIGS-6	Habitat	thermal soil	TAS [1]
MIGS-15	Biotic relationship	free living	NAS
MIGS-14	Pathogenicity	none	NAS
	Biosafety level	1	TAS [24]
	Isolation	65°C hot extreme thermal soil in small depression site	TAS [1]
MIGS-4	Geographic location	Near Rabbit Creek and Firehole River in Yellowstone National Park, Wyoming, USA	TAS [1]
MIGS-5	Sample collection time	June 1998	NAS
MIGS-4.1	Latitude	44.394	NAS
MIGS-4.2	Longitude	-110.568	NAS
MIGS-4.3	Depth	0 m, soil surface	TAS [1]
MIGS-4.4	Altitude	not reported	

Evidence codes - IDA: Inferred from Direct Assay (first time in publication); TAS: Traceable Author Statement (i.e., a direct report exists in the literature); NAS: Non-traceable Author Statement (i.e., not directly observed for the living, isolated sample, but based on a generally accepted property for the species, or anecdotal evidence). These evidence codes are from of the Gene Ontology project [25]. If the evidence code is IDA, then the property was directly observed by one of the authors or an expert mentioned in the acknowledgements

### Chemotaxonomy

Murein is present in large amounts, which is consistent with the observed thick (approximately 34 nm) cell walls with a muramic acid content similar to that of *Bacillus subtilis* [1]. The muramic acid content of strain YNP1^T^ was roughly one quarter of that measured for *B. subtilis*) but almost 40-fold greater than in *E. coli* [1]. Lipopolysaccharide (LPS) was not detected [1]. Major fatty acids were dominated by straight and branched chain saturated acids: C_18:0_ (27.0%); iso-C_17:0_ (11.6%); iso-C_19:0_ (12.9%); anteiso-C_18:0_ (12.5%); C_20:0_ (16.5%) and C_19:0_ (6.6%). The pink pigment associated with strain YNP1^T^ exhibited a significant absorption at wavelengths 267, 326, 399, 483, 511, and 549 nm [1].

## Genome sequencing and annotation

### Genome project history

This organism was selected for sequencing on the basis of its phylogenetic position [26], and is part of the *** G****enomic* *** E****ncyclopedia of* *** B****acteria and* *** A****rchaea * project [27]. The genome project is deposited in the Genome OnLine Database [15] and the complete genome sequence is deposited in GenBank. Sequencing, finishing and annotation were performed by the DOE Joint Genome Institute (JGI). A summary of the project information is shown in Table 2.

**Table 2:** Genome sequencing project information

**MIGS ID**	**Property**	**Term**
MIGS-31	Finishing quality	Finished
MIGS-28	Libraries used	Two genomic libraries: one Sanger 8 kb pMCL200 library, one 454 pyrosequence standard library
MIGS-29	Sequencing platforms	ABI3730, Illumina GAii, 454 GS FLX
MIGS-31.2	Sequencing coverage	9.5 × Sanger; 31.8 x pyrosequence
MIGS-30	Assemblers	Newbler version 1.1.02.15, phrap
MIGS-32	Gene calling method	Prodigal 1.4, GenePRIMP
	INSDC ID	CP001825 (chromosome 1) CP001826 (chromosome 2)
	Genbank Date of Release	November 23 and 25, 2009
	GOLD ID	Gc01150
	NCBI project ID	29523
	Database: IMG-GEBA	2501533217
MIGS-13	Source material identifier	ATCC BAA-798
	Project relevance	Tree of Life, GEBA

## Growth conditions and DNA isolation

*T. terrenum* strain YNP1^T^, ATCC BAA-798, was grown in ATCC medium 1981 (M-R2A medium) [28] at 60°C. The culture used to prepare genomic DNA (gDNA) for sequencing was only two transfers from the original deposit. The purity of the culture was determined by growth on general maintenance media under both aerobic and anaerobic conditions. Cells where harvested after 24 hours by centrifugation and gDNA was extracted from lysozyme-treated cells using CTAB and phenol-chloroform. The purity, quality and size of the bulk gDNA preparation was assessed according to DOE-JGI guidelines. Amplification and partial sequencing of the 16S rRNA gene confirmed the isolate as ‘*T. terrenum’*. The quantity of the DNA was determined on a 1% agarose gel using mass markers of known concentration supplied by JGI. The average fragment size of the purified gDNA determined to be ~43kb by pulsed-field gel electrophoresis.

### Genome sequencing and assembly

The genome was sequenced using a combination of Sanger and 454 sequencing platforms. All general aspects of library construction and sequencing can be found at the JGI website (http://www.jgi.doe.gov/). Pyrosequencing reads were assembled using the Newbler assembler version 1.1.02.15 (Roche). Large Newbler contigs were broken into 3,926 overlapping fragments of 1,000 bp and entered into assembly as pseudo-reads. The sequences were assigned quality scores based on Newbler consensus q-scores with modifications to account for overlap redundancy and adjust inflated q-scores. A hybrid 454/Sanger assembly was made using the parallel phrap assembler (High Performance Software, LLC). Possible misassemblies were corrected with Dupfinisher or transposon bombing of bridging clones [29]. A total of 432 Sanger finishing reads were produced to close gaps, to resolve repetitive regions, and to raise the quality of the finished sequence. Illumina reads were used to improve the final consensus quality using an in-house developed tool (the Polisher [30]). The error rate of the completed genome sequence is less than 1 in 100,000. Together, the combination of the Sanger and 454 sequencing platforms provided 10.0× coverage of the genome. The final assembly contains 32,920 Sanger reads.

### Genome annotation

Genes were identified using Prodigal [31] as part of the Oak Ridge National Laboratory genome annotation pipeline, followed by a round of manual curation using the JGI GenePRIMP pipeline [32]. The predicted CDSs were translated and used to search the National Center for Biotechnology Information (NCBI) nonredundant database, UniProt, TIGRFam, Pfam, PRIAM, KEGG, COG, and InterPro databases. Additional gene prediction analysis and functional annotation was performed within the Integrated Microbial Genomes - Expert Review (IMG-ER) platform [33].

### Genome properties

**Figure 3:**
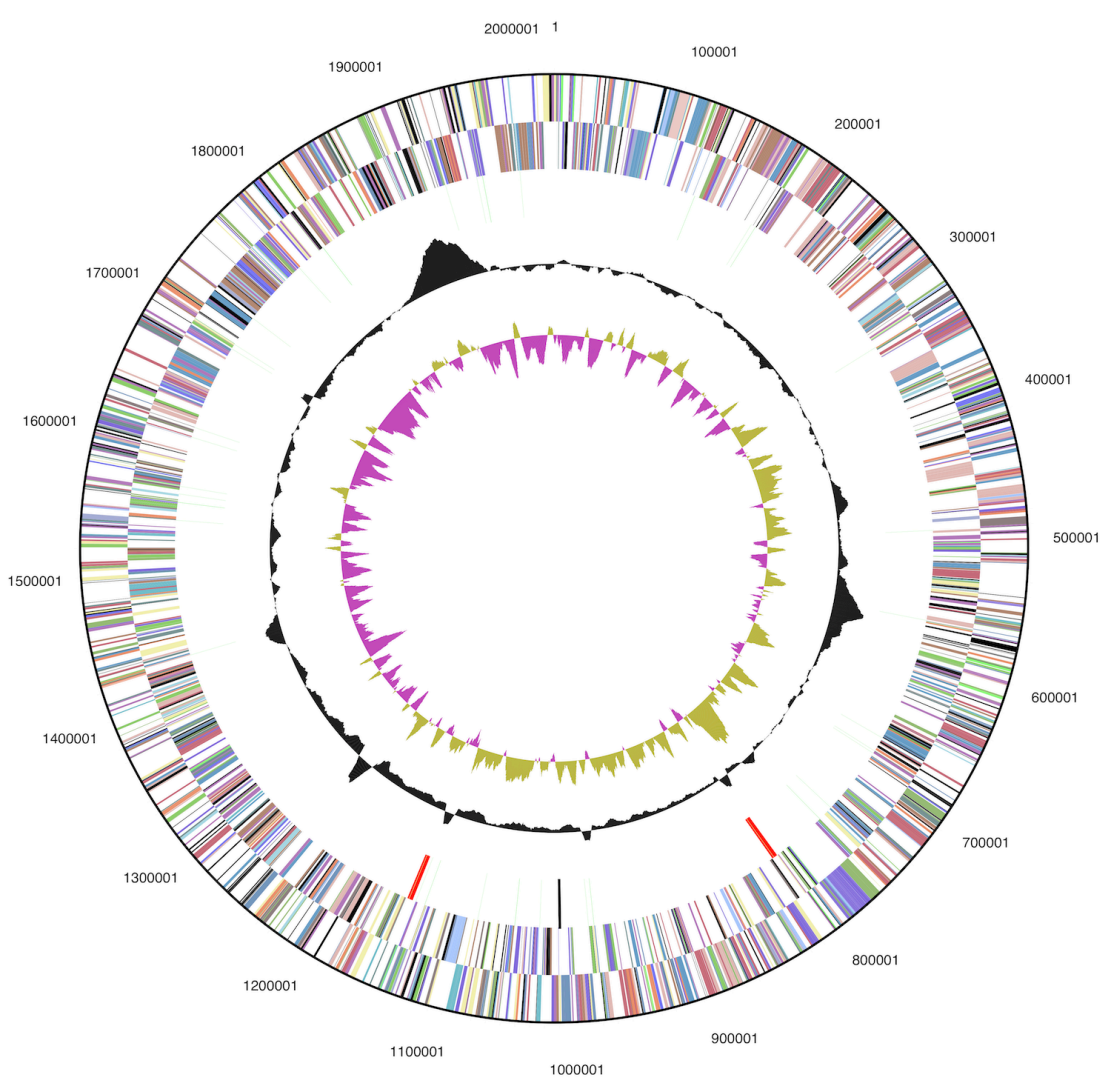
Graphical circular map of the 2Mb low G+C chromosome 1. From outside to the center: Genes on forward strand (color by COG categories), Genes on reverse strand (color by COG categories), RNA genes (tRNAs green, rRNAs red, other RNAs black), GC content, GC skew.

**Figure 4:**
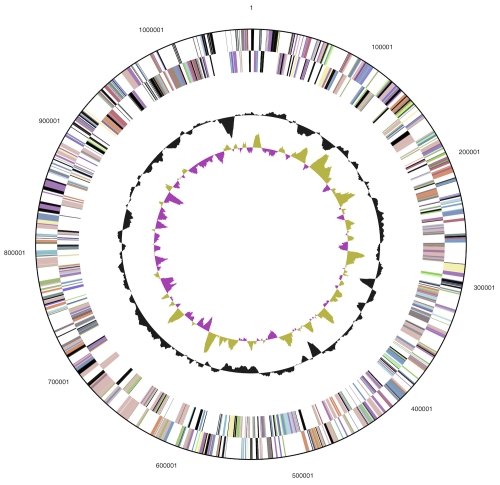
Graphical circular map of the 1 Mb high-G+C chromosome 2. From outside to the center: Genes on forward strand (color by COG categories), Genes on reverse strand (color by COG categories), RNA genes (tRNAs green, rRNAs red, other RNAs black), GC content, GC skew.

The genome consists of two chromosomes: the low G+C (48%) 2,026,947 bp long chromosome 1, and the high G+C (64%) 1,074,634 bp long chromosome 2 (Table 3,  Figure 3, Figure 4). Of the 2,930 genes predicted (1,935 on chromosome 1 and 995 on chromosome 2), 2,872 were protein-coding genes, and 58 RNAs; forty one pseudogenes were also identified. The majority of the protein-coding genes (73.4%) were assigned a putative function while the remaining ones were annotated as hypothetical proteins. The distribution of genes into COGs functional categories is presented in Table 4.

**Table 3:** Genome Statistics

**Attribute**	**Value**	**% of Total**
Genome size (bp)	3,101,581	100.00%
DNA coding region (bp)	2,825,726	91.11%
DNA G+C content (bp)	1,659,573	53.51%
Number of replicons	2	
Extrachromosomal elements	0	
Total genes	2,930	100.00%
RNA genes	58	1.98%
rRNA operons	2	
Protein-coding genes	2,872	98.02%
Pseudo genes	41	1.40%
Genes with function prediction	2,151	73.41%
Genes in paralog clusters	439	14.98%
Genes assigned to COGs	2,223	75.78%
Genes assigned Pfam domains	2,308	78.77%
Genes with signal peptides	573	19.56%
Genes with transmembrane helices	777	26.52%
CRISPR repeats	6	

**Table 4:** Number of genes associated with the general COG functional categories

**Code**	**Value**	**%age**	**Description**
J	139	5.6	Translation, ribosomal structure and biogenesis
A	0	0.0	RNA processing and modification
K	158	6.4	Transcription
L	110	4.5	Replication, recombination and repair
B	1	0.0	Chromatin structure and dynamics
D	18	0.7	Cell cycle control, cell division, chromosome partitioning
Y	0	0.0	Nuclear structure
V	56	2.3	Defense mechanisms
T	106	4.3	Signal transduction mechanisms
M	131	5.3	Cell wall/membrane/envelope biogenesis
N	2	0.1	Cell motility
Z	0	0.0	Cytoskeleton
W	0	0.0	Extracellular structures
U	35	1.4	Intracellular trafficking and secretion, and vesicular transport
O	105	4.3	Posttranslational modification, protein turnover, chaperones
C	160	6.5	Energy production and conversion
G	325	13.2	Carbohydrate transport and metabolism
E	206	8.4	Amino acid transport and metabolism
F	60	2.4	Nucleotide transport and metabolism
H	128	5.2	Coenzyme transport and metabolism
I	67	2.7	Lipid transport and metabolism
P	134	5.4	Inorganic ion transport and metabolism
Q	44	1.8	Secondary metabolites biosynthesis, transport and catabolism
R	316	12.8	General function prediction only
S	162	6.6	Function unknown
-	707	24.1	Not in COGs
